# *In-vivo* Anti-inflammatory Activity of* Hydrocotyle umbellata* L. aerial parts and Isolation of the Main Phytochemicals 

**DOI:** 10.22037/ijpr.2020.1101154

**Published:** 2020

**Authors:** Sherif A. Hamdy, Esther T. Menze, Hala M. El Hefnawy, Shadia M. Azzam, Elsayed A. Aboutabl

**Affiliations:** a *Department of Pharmacognosy, Faculty of Pharmacy, Cairo University, Kasr El-Aini St., Cairo 11562, Egypt. *; b *Department of Pharmacology and Toxicology, Faculty of Pharmacy, Ain Shams University, Cairo, Egypt.*

**Keywords:** Hydrocotyle umbellata L., Anti-inflammatory, IL-6, PGE_2_, Quercetin, Neochlorogenic acid

## Abstract

*Hydrocotyle umbellata* L. (Family Araliaceae) populary known as Acaricoba, is indicated in folk medicine for treatment of several inflammatory disorders. The goal of the present study is to evaluate the anti-inflammatory activity of the defatted ethanolic extract (DEE) of the aerial parts using carrageenan-induced rat paw oedema method. The levels of the pro-inflammatory cytokine interleukin-6 (IL-6) and the inflammatory mediator prostaglandin E_2_ (PGE_2_) were assessed using ELISA. The DEE at dose level 100 mg/kg showed significant decrease in oedema volume after 2 and 3 h, equivalent to 70.75% and 95.92% of the activity of the standard anti-inflammatory indomethacin, respectively. DEE significantly decreased the concentrations of the excessively produced IL-6 and PGE_2_ (24 ± 2.1 and 2374 ± 87 pg/mL compared to 16 ± 2 and 2419 ± 95 pg/mL induced by indomethacin). Chemical investigation was carried out to isolate and identify the bioactive compounds that might be responsible for this activity. The total phenolic (79.28 ± 0.1 mg) and total flavonoid (57.99 ± 0.1 mg) contents of the DEE were quantified spectrophotometricaly and expressed as gallic acid and rutin equivalents/g dry weight, respectively. The DEE was subjected to further fractionation using solvents of increasing polarities. Purification of the ethyl acetate fraction using different chromatographic techniques led to the isolation of five compounds, which were identified through 1D and 2D and UV/Vis spectral data. The five compounds were: quercetin, avicularin, quercitrin, hyperoside, and neochlorogenic acid. Several biological studies confirmed the important role of caffeoyl quinic acid and quercetin derivatives as anti-inflammatory bioactive compounds.

## Introduction

Non-steroidal anti-inflammatory drugs (NSAIDS) are commonly used for treatment of pain and many inflammatory disorders (1). Because of the side effects they produce, like ulcer and renal disorder, many people rely on the use of herbal medicine as a source of bioactive metabolites with less side effect (2, 3).

Genus *Hydrocotyle* is a genus of creeping perennial herbs of wet places, belongs to the Araliaceae family, distributed throughout the tropical and subtropical regions (4). Lin *et al*., reported the use of *Hydrocotyle* species in Taiwan folk medicine in treatment of many inflammatory diseases, dysentery, zoster, and menstrual pain (5). The main secondary metabolites detected in most *Hydrocotyle* species were flavonoid glycosides and triterpenoids, to which several biological activites of *Hydrocotyle* species could be attributed (6-10).

*Hydrocotyle umbellata* L. is a creeper herbaceous plant, widely distributed in the Americas (11), characterized by long cylindrical petiole attached to the center of peltate-shaped leaves. The flowers are small, white-coloured, and clustered on long peduncle that exceeds the length of the leaf petiole (12, 13)*.* It has a great interest in folk medicine because of its anti-inflammatory, anxiolytic, and memory stimulant activities (5, 14). Florentino *et al*., and Rocha *et al*., reported the analgesic, anti-anflammatory and anxiolytic activities of the ethanolic extract of *H. umbellata* L. underground parts (11, 15). Due to the interesting medicinal value of *H.* *umbellata *L. and lack of reports concerning the biological and chemical investigation of *H.* *umbellata *L. aerial parts, this study was designed to evaluate the anti-inflammatory activity of the DEE of *H.* *umbellata *L. aerial parts, followed by further chemical analysis to isolate and characterize its major phytoconstituents that might be correlated to its biological activity.

## Experimental

*Plant material*

The Samples of *H. umbellata *L*. *aerial parts (leaves and flowers) were collected in May 2014 from El-Orman Botanical Garden, Giza, Egypt and were cultivated in Experimental Station for Aromatic and Medicinal plants, Faculty of Pharmacy, Cairo University, Giza, Egypt. The plant identity was authenticated by Eng. Threase Labib, consultant in Orman Garden and National Gene Bank, Ministry of Agriculture and confirmed by Dr. Mohammed El-Gebaly, senior taxonomist at National Research Center. A voucher specimen (No. 17-8-2016) was kept at the Herbarium of Pharmacognosy Department, Faculty of Pharmacy, Cairo University.

*Preparation of DEE and different extractives*

The air-dried powdered aerial parts (2.3 kg) were defatted using *n*-hexane**, **then extracted with 70% ethanol till exhaustion. The combined ethanolic extracts were evaporated under reduced pressure to dryness yielding 164.2 g dry residue of DEE (7.1%). An aliquot of this dry residue (110 g) was suspended in water (400 mL) and successively partitioned with methylene chloride, ethyl acetate, and *n*-butanol saturated with water. Each fraction was evaporated under reduced pressure to dryness yielding 4.4 (4%), 11 (10%) and 18.7 (17%) g of each fraction, respectively. The fractions were kept in tightly closed glass containers and kept in a desiccator for phytochemical and bioactivity studies.

*Chemicals for anti-inflammatory activity, total phenolics and total flavonoids*

 Inflammatory-grade carrageenan was purchased from FMC (Rockland, ME). Indomethacin was purchased from Sigma-Aldrich (Taufkirchen, Germany). IL-6 was purchased from (Boster Biological Technology Co., Inc., Valley Ave Pleasanton, CA). PGE_2_ was purchased from (Research and Development Systems, MN, USA). Folin-Ciocalteu obtained from Loba-Chemie (Mumbai, India). All other chemicals and standards were purchased from Sigma–Aldrich (St Louis, MO, USA).

*Experimental animals*

Adult male Wistar rats obtained from the animal house colony, National Research Center, Giza, Egypt, weighing (150-200 g) were used for determentaion of LD_50_ and anti-inflammatory study. They were housed at a temperature 23 ± 2 ºC and 55 ± 5% humidity with 12 h light/dark cycle, with free access to standard food pellets composed of vitamins mixture (1%), minerals mixture (4%), corn oil (10%), sucrose (20%), cellulose (0.2%), casein (10.5%), and starch (54.3%). Water was supplied *ad* *libitum*. The experimental protocol followed the Institutional Animal Ethical Committee of the National Research Centre.

*Median lethal dose LD*_50_

LD_50_ of the DEE of *H. umbellata* L. aerial parts was performed as per OECD-425 guidelines (16). Five Wistar albino rats of uniform weight were selected. One animal was fasted overnight with free access to drinking water. They were given 2000 mg/kg of the test extract (DEE) and observed for 24 h for mortality. The animal survived and then four additional animals were tested sequentially, so that a total of five animals were tested. All the animals were observed closely for 24 h and daily for 14 days, no mortality was observed. 

*In-vivo anti-inflammatory activity using carrageenan-induced rat paw oedema*

It was carried out according to the carrageenan-induced rat paw oedema method (17). Four groups of adult male albino Wistar rats were used (1-4), each of 6 animals. Groups 1 and 2 received the vehicle (5% carboxymethylcellulose). Animals of group 3 were given indomethacin orally in a dose of 10 mg/kg b.wt. as a standard anti-inflammatory drug, and the remaining group was orally received the DEE in a concentration of 100 mg/kg b.wt. One hour later, group 1 received 0.05 mL of saline, whereas groups 2–4 received 0.05 mL of carrageenan (1% solution in saline) subcutaneously on the plantar surface of the right hind paw. The rats were sacrificed 3 hr after the induction of inflammation. The right hind paw volume was measured immediately after carrageenan injection by water displacement using a plethysmometer (model 7140, Ugo Basile, Comerio, Italy). The paw volume was re-measured 1, 2, and 3 h after injection of carrageenan (18).

The mean response for increase in the paw oedema after acute inflammation was calculated: 

Oedema% = Weight of the right paw – Weight of the left Paw/Weight of the left paw × 100 

Furthermore, the percentage of inhibition in the mean of the treated group in comparison with the control non-treated group was estimated and calculated according to the following equation:

Inhibition% = Paw edema of control - Paw edema of treated/Paw edema of control × 100

*Measurment of IL-6 and PGE*_2_ *levels in the rat paw*

Right hind paws were removed. A volume of 0.1 mL of saline containing 10µM indomethacin was injected to aid removal of the eicosanoid-containing fluid and to stop further production of IL-6 and PGE_2__._ Paws were incised with a scalpel and suspended off the bottom of polypropylene tubes with Eppendorf pipette tips to facilitate drainage of the inflammatory exudates. For the purpose of the removal of the inflammatory exudates, the paws were centrifuged at 4000 rpm for 15 min at 4 °C and the supernatants were separated and assayed. IL-6 and PGE_2_ were quantified in the collected exudates using enzyme- linked immunosorbent assay kits. Both assays are based on the sandwich technique, in which specific antibodies to IL-6 or PGE_2_ were pre-coated on to 96-well plate. The specific detection antibodies were biotinylated. The test samples and biotinylated detection antibodies were added sequentially followed by washing. Avidin-biotin-Peroxidase complex was added and unbound conjugates were washed. A substrate solution is added to the wells to determine the bound enzyme activity. The colour development is stopped, and the absorbance is read at 450 nm using an ELISA microplate reader (ChroMate-4300, Palm City, FL). The quantifications of IL-6 and PGE_2_ were done according to the instructions of ELISA kits.

*Statistical analysis*

The data were expressed as the means ± SEM. The differences between groups were tested by one-way analyses of variance followed by the Tukey post hoc test. All statistical analyses were performed using Graph Pad Instat software version 3 (ISI software, Marinadel Rey, CA). The probability of* p *< 0.05 was considered statistically significant.

*Toatal phenolics and total flavonoids*

Spectrophotometric determination of total phenolic content was carried out using the Folin-Ciocalteu colourimetric method (19), while the total flavonoids were determined using AlCl_3 _colourimetric assay (20).

*Chemicals for phytochemical investigation*

Authentic reference samples used in co-chromatography were purchased from Sigma Chemical Co. (St. Louis, MO, USA). Silica gel H 60 for vacuum liquid chromatography (VLC) was purchased from E-Merck (Darmstadt, Germany). Silica gel 60 and silica gel RP-18 for column chromatography were obtained from Fluka, Sigma-Aldrich Chemicals, Germany. Sephadex LH-20 was purchased from Pharmacia Fine Chemicals AB (Uppsala, Sweden). Precoated TLC plates and silica gel 60 F 254 was obtained from Fluka, Sigma-Aldrich Chemicals, Germany. The chromatograms were visualized under UV light (at 254 and 366 nm) before and after exposure to ammonia vapor, as well as spraying with *p*-anisaldehyde/sulfuric acid (21) and/or natural products/polyethylene glycol (NP/PEG) spray reagents (22). Shift reagents for UV spectroscopy according to the published procedures (23) and the chemicals used were obtained from E-Merck, Darmstadt, Germany.

*Apparatus and equipment* *for phytochemical investigation*

Schimadzu double beam spectrophotometer (UV-1650, Japan) was utilized for determination of UV shifts of flavonoids. NMR spectra were recorded at 400 (^1^H) and 100 MHz (^13^C) on a Bruker NMR-spectrometer, Japan. The NMR spectra were recorded in deuterated CD_3_OD and DMSO using TMS as an internal standard. 

*Phytochemical investigation of the ethyl acetate fraction*

The ethyl acetate fraction (10 g) was chromatographed on a vacuum liquid column (VLC) (12.5 × 7 cm), packed with 190 g silica gel H 60. Gradient elution was performed starting with methylene chloride and gradually increasing the polarity with ethyl acetate by 10% increments till 100% ethyl acetate followed by increasing the polarity with methanol in 5% increments till 100% pure methanol. The fractions were collected (300 mL each) and monitored by TLC and the similar ones were pooled yielding five fractions designated as fractions I-V. Fraction I (eluted with 40-70% EtOAc in CH_2_Cl_2_) was purified on sephadex LH-20 column using methanol: water (50:50) v/v for elution to yield 100 mg of yellow microcrystals (C_1_). Fraction II (eluted with 5% methanol in EtOAc) was chromatographed on sephadex LH-20 column using methanol: water (50:50) v/v for elution to yield 50mg of a yellow microcrystals (C_2_). Fraction III (eluted with 10-15% methanol in EtOAc) was chromatographed on a sephadex LH-20 column using methanol: water (50:50) v/v for elution to yield 40 mg of yellow microcrystals (C_3_). Fraction IV (eluted with 20-25% methanol in EtOAc) was rechromatographed on a VLC silica gel RP-C18 column (2 × 20 cm) using methanol: water for elution with gradual 5% increments of methanol. Subfractions eluted with 15-20% methanol in water were pooled and rechromatographed on a sephadex LH-20 column using methanol: water (50:50) v/v for elution to yield 70 mg of a yellow microcrystals (C_4_). Fraction V (eluted with 30-40% methanol in EtOAc) was chromatographed on a sephadex LH-20 column using methanol: water (50:50) v/v for elution. Fractions of 5 mL were collected and similar fractions were pooled together yielding one subfraction, which was further purified on another sephadex LH-20 column using methanol: water (50:50) v/v for elution to yield 60 mg of a white amorphous powder (C_5_). 

## Results and Discussion

*In-vivo anti-inflammatory activity*

The inflammation induced by carrageenan is acute, non-immune and highly reproducible. The signs of inflammation including oedema, hyperalgesia and erythema develop immediately after subcutaneous injection of carrageenan due to the action of pro-inflammatory agents (24). Carrageenan stimulates the release of the pro-inflammatory cytokine interleukin 6 (IL-6) (18) and the inflammatory mediator prostaglandin E2 (PGE_2_), both IL-6 and PGE_2_ involved in initiation and amplification of the inflammation (18, 25). Therefore, inhibition of IL-6 and PGE_2_ production may serve to prevent or suppress a variety of inflammatory diseases such as hepatitis, cystitis, and rheumatoid arthritis (26). The inflammatory response is usually quantified by increase in paw size (oedema) that reaches its maximal level within 3 h (18). The intraplantar injection of carrageenan into adult male rats resulted in a severe inflammation and significant increase in the mean volume of the right hind paw compared to that of the untreated paws after 2 and 3 h of injection (Table 1). Pretreatment with DEE at a dose level 100 mg/kg showed a highly significant anti-inflammatory activity after 2 h and 3 h (70.73%) and (95.92%), respectively, with maximum activity after 3 h compared to standard anti-inflammatory indomethacin (Table 1). Injection of carrageenan into the rat hind paw induced a significant increase in the hind paw IL-6 and PGE_2_ concentrations, 3 h after injection, reaching 757% and 165% of control group, respectively**.** Pretreatment with DEE at a dose level 100 mg/kg caused a significant reduction in the high concentration of IL-6and PGE_2_ by 50% (about 75% of the activity of indomethacin) and 24.4% (slightly potent than indomethacin), (Tables 2 and 3, respectively).

*Total phenolics, total flavonoids and phytochemical investigation*

Available reports claimed that secondary metabolites of phenolic nature including flavonoids are responsible for several biological activities including anti-inflammatory activity, so it was a matter of interest to estimate the total phenolics and total flavonoids contents in addition to isolation and characterization of the major phenolic compounds accumulated in *H. umbellata* L. aerial parts (27, 28, 29, 30, and 31).

The total phenolic and total flavonoid contents were 79.282 ± 0.1 mg GAE/g and 57.999 ± 0.1 mg rutin equivalent/g dry weights, respectively. 

**Figure 1:**
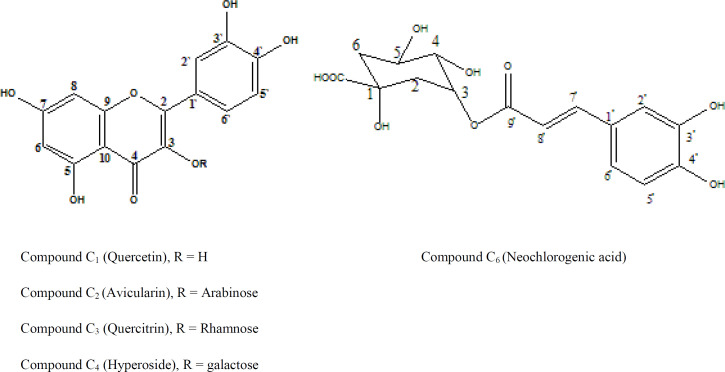
Structure of the isolated compounds

Chemical investigation of the ethyl acetate fraction resulted in isolation of four flavonoids and one phenolic acid designated as 1-5*. *The structures of the isolated compounds are shown in Figure 1 and their spectral data are recorded in Tables 4-7. 

Compound** C**_1 _was identified as quercetin through its ^1^H-NMR spectrum signals, which is characteristic for quercetin nucleus and by co-chromatography with authentic sample. Compounds** 2, 3, **and** 4** were identified as quercetin-3-*O*-monosaccharides. The structure of each compound was elucidated from its respective ^1^H and ^13^C-NMR spectral data. The upfield shift of their C-3 resonances in addition to their UV spectral pattern in methanol and after addition of different UV shift reagents confirmed the attachment of the sugar to C-3. Using co-chromatography with authentic samples and upon a comparison of the spectral data with the available literature compounds **3, 4, **and **5** were identified as quercetin-3-*O*-*α*-arabinofuranoside (Avicularin), quercetin-3-*O*-*α*- rhamnopyranoside (Quercitrin) and quercetin-3-*O*-*β-*galactopyranoside (hyperoside), respectively (32-34). Compound** C**_5_ exihibited a sky blue colour in UV, which turns to greenish yellow colour on exposure to ammonia vapour and spraying with AlCl_3_. The UV spectral data of compound C_5_ in methanol indicated that it could be a hydroxycinnamic acid derivative (35). ^1^H-NMR spectrum of compound C_5_ showed the characteristic signals for caffeic acid. Also, the protons of a quinic acid moiety could be observed with a doublet at δ 1.73 (*J* = 12.9 Hz) assigned to H-6 _ax_, a doublet of doublet at δ 1.90 ppm (*J* = 13.8, 10.8 Hz) assigned to H-6 _eq_ and a multiplet at 2.08 ppm integrated as two protons assigned to H-2 _ax_ and H-2 _eq_, respectively. A broad singlet at δ 3.58 and a doublet at δ 3.62 (*J* = 7.08 Hz) were assigned to H-4 and H-5, respectively. The acylation of quinic acid by caffeic acid at the OH on C-3 was confirmed by the downfield shift of H-3 which appeared at δ 5.28 ppm (36). The assignment of quinic acid moiety protons was determined using ^1^H-^1^H COSY. 

On the basis of the previous discussion and published data and by direct comparison with an authentic, compound C_5_ was identified as neochlorogenic acid (3-*O*-Caffoeylquinic acid) (37, 38)*.*

**Table 1:** Acute anti-inflammatory effect of the DEE of *Hydrocotyle umbellata* L. aerial parts

**Group**	**Dose**	**1 (h)**		**2 (h)**		**3 (h)**	
		**Paw volume (mL)**	**Oedema Inhibition (%)**	**Paw volume (mL)**	**Oedema Inhibition (%)**	**Paw volume (mL)**	**Oedema Inhibition (%)**
Control	^_^	0.4 ± 0.01	^_^	0.39 ± 0.01	^_^	0.41 ± 0.01	^_^
Carrageenan	^_^	0.64^a^ ± 0.03	^_^	0.65^a^ ± 0.02	^_^	0.74^a^ ± 0.02	^_^
Indomethacin	10 (mg/kg)	0.49 ± 0.01	23.4	0.41^b^ ± 0.015	36.9	0.5^b^ ± 0.02	32.4
DEE	100 (mg/kg)	0.56 ± 0.02	12.5	0.48^b^ ± 0.03	26.1	0.51^b^ ± 0.02	31.08

^a^statistically significant from the control group at *p* > 0.05. ^b^statistically significant from the carrageenan alone-treated group at *p* > 0.05. Statistical analysis was carried out using repeated one-way ANOVA test followed by Tukey test for multiple comparisons.

**Table 2:** Effect of oral administration of the DEE of *Hydrocotyle umbellata* L. aerial parts on the level of IL-6 in carrageenan-induced rat paw oedema model

**Group**	**IL-6 conc. (pg/mL) (mean ± SEM)**
Control	5.6 ± 0.51
Carrageenan	48^a^ ± 1.5
Indomethacin (10 mg/kg)	16^b^ ± 2
DEE (100 mg/kg)	24^b^ ± 2.1

^a^statistically significant from the control group at *p* > 0.05. ^b^statistically significant from the carrageenan alone-treated group at *p* > 0.05. Statistical analysis was carried out using repeated one-way ANOVA test followed by Tukey test for multiple comparisons.

**Table 3:** Effect of oral administration of the DEE of *Hydrocotyle umbellata* L. aerial parts on the level of PGE_2_ in carrageenan-induced rat paw oedema model

**Group**	**PGE-2 conc. (pg/mL) (mean ± SEM)**
Control	1183 ± 128
Carrageenan	3141^a^ ± 106
Indomethacin (10 mg/kg)	2419^a,b ^± 95
DEE (100 mg/kg)	2374^b^ ± 87

^a^statistically significant from the control group at *p* > 0.05. ^b^statistically significant from the carrageenan alone-treated group at *p* > 0.05. Statistical analysis was carried out using repeated one-way ANOVA test followed by Tukey test for multiple comparisons.

**Table 4:** UV-shifts of isolated flavonoids and phenolic acid

	**MeOH**	**Na Methoxide**	**AlCl**_3_	**AlCl**_3_ **/HCl**	**Na Acetate**	**Na Acetate/Boric acid**
C_1_ quercetin	255, 301sh, 368	247sh, 330, 406	271, 305sh, 333, 458	267,301sh, 352sh, 429	268, 329sh, 390	261, 303sh, 387
C_2_ avicularin	256, 269sh, 300sh, 358	271, 325sh, 406	273, 307 sh, 335 sh, 434	269, 303sh, 362sh, 401	274, 323sh, 390	262, 301sh, 376
C_3_ quercitrin	255, 270sh, 301sh, 349	271, 327sh,400	276, 304sh,332,429	272, 303sh,356,400	271,329sh,377	260,292sh,368
C_4_ hyperoside	257, 269sh, 299sh, 362	272, 327sh, 410	275, 305sh, 335sh, 437	268, 300sh, 360, 405	274, 324sh, 390	262, 297sh, 376
C_5_ neochlorogenic acid	290, 326	No change				

**Table 5:** ^1^H-NMR of the isolated flavonoids

**Position**	**C**_1_ **(400 MHz, CD**_3_ **OD)**	**C**_2_ **(400 MHz, DMSO)**	**C**_3_ **(400 MHz, DMSO)**	**C**_4_ **(400 MHz, CD**_3_ **OD)**
6	6.08 (1H, *d*, *J*_6.8_ = 2 Hz)	6.12 (1H, *br.s*)	6.21 (1H, *d*, *J*_6.8_ = 1.96) Hz)	6.09 (1H, *br.s*,)
8	6.29 (1H, *d*, *J*_8.6_ = 2 Hz)	6.34 (1H, *br.s)*	6.39 (1H, *d*, *J*_8.6_ = 1.96 Hz)	6.29 (1H, *br.s*)
2'	7.63 (1H, *d*, *J*_2'.6'_ = 2 Hz)	7.52 (1H, *d*, *J*_2'.6'_ = 2.1 Hz)	7.31 (1H, *d*,* J*_2'.6'_ = 2 Hz)	7.74 (1H, *br.s)*
5'	6.78 (1H, *d*, *J*_5'.6'_ = 8.5 Hz)	6.82 (1H, *d*, *J*_5'.6'_ = 8.4 Hz)	6.86 (1H, *d*,* J*_5'.6'_ = 8.3 Hz)	6.76 (1H, *d*, *J*_5'.6'_ = 8.48 Hz)
6'	7.53 (1H, *dd*, *J*_6'. 2'_ = 2, *J*_6'. 5'_ = 8.5 Hz)	7.49 (1H, *dd J*_6'. 2'_ = 2.2 *J*_6'. 5'_ = 8.2 Hz)	7.27 (1H, *dd*, *J*_6'. 2'_ = 2, *J*_6'. 5'_ = 8.3 Hz, H-6')	7.48 (1H, *dd*, *J*_6'. 2'_ = 2, *J*_6'. 5'_ = 8 Hz)
Sugar protons		5.57 (1H, *br. s*, H-1''), 3.28 - 4.17	5.26 (1H, *br.s*, H-1'), 0.82 (3H, *d*, *J *= 5.92 Hz, H-6''), 3.1-4.2	5.05 (1H, *d*, *J *= 7.8 Hz, H-1'), 3.36 - 3.75

**Table 6.** ^1^H-NMR of the isolated phenolic acid (**C**_5_)

**Position**	**C**_6_ **(400 MHz, CD**_3_ **OD)** **neochlorogenic acid**
3	5.28
4	3.58 (*br.s*),
5	3.62 (*J* = 7.08 Hz)
2'	6.9 (*br.s*),
5'	6.6 (*d*, *J *= 8 Hz)
6'	6.8 (*d*, *J* = 8 Hz)
7'	δ 7.4 (d, *J* = 15.8 Hz)
8'	6.1 (d, *J* = 18.9 Hz)
2ax, eq	2.08 (2H, m)
6ax	1.73 (*J* = 12.9 Hz)
6eq	1.90 (*J* = 13.8, 10.8 Hz)

**Table 7:** ^13^C-NMR of the isolated flavonoids

	**C**_2_ **(100 MHz, DMSO)**	C_3_ **(100 MHz, DMSO)**	C_4_ **(100 MHz, CD**_3_**OD)**
2	157.1	157.03	157.34
3	133.6	134.8	134.49
4	178	178.4	178.13
5	161.71	161.75	161.79
6	99.8	99.04	98.75
7	165.20	164.65	165.13
8	94.35	94.09	93.60
9	158.17	157.76	157.34
10	103.62	104.54	104.31
1'	121.84	121.51	121.73
2'	116.01	116.04	116.31
3'	145.98	145.49	144.35
4'	149.71	148.88	148.73
5'	115.64	115.80	114.80
6'	121.40	121.51	121.49
Sugar protons	108.36 (C1'') 77.20 (C2''), 82.49 (C3''), 86.45 (C4''), 61.26 (C-5'')	102.46 (C-1''), 70.8 (C-2''), 71.04 (C-3''), 71.64 (C-4''), 70.58 (C-5''), 17.85 (C-6'').	103.80 (C-1''), 71.81 (C-2''), 73.63 (C-3''), 68.93 (C-4''), 75.98 (C-5''), 60.62 (C-6'')

## Conclusion

 The DEE of *H. umbellata* L. aerial parts at a dose level 100 mg/kg showed significant anti-inflammatory activity using carrageenan-induced rat paw oedema model with significant reduction in IL-6 and PGE-2 high concentrations induced by carrageenan. Four flavonoids of quercetin nucleus and neochlorogenic acid were successfully isolated and identified from the ethyl acetate fraction of *H. umbellata *L. aerial parts. The isolated flavonoids and neochlorogenic acid were previously reported to exhibit anti-inflammatory activity (39, 40, 41, 42, 43 and 44). To the best of our knowledge, this is the first time of avicularin to be isolated from genus *Hydrocotyle*, while quercitrin and neochlorogenic acid are isolated for the first time from *H. umbellata *L. 

## References

[1] Conforti F, Sosa S, Marrelli M, Menichini F, Statti GA, Uzunov D, Menichini F (2009). The protective ability of Mediterranean dietary plants against the oxidative damage: the role of radical oxygen species in inflammation and the polyphenol, flavonoid and sterol contents. Food Chem..

[2] Robert A (1976). Antisecretory, antiulcer, cytoprotective and diarrheogenic properties of prostaglandins. Adv. Prostaglandin Thromboxane Res..

[3] Gupta M, Mazumder UK, Gomathi P, Selvan VT (2006). Anti-inflammatory evaluation of leaves of Plumeria acuminate. BMC Complement. Altern. Med..

[4] Karuppusamy S, Ali MA, Rajasekaran K, Lee J, Kim SY, Pandey AK, Al-Hemaid FM (2014). A new species of Hydrocotyle L (Araliaceae) from India. Bangladesh J. Plant Taxon..

[5] Lin IH, Chang YS, Chen IS, Hsieh WC (2003). The Catologue of Medicinal PlantResourses in Taiwan. Committee on Chinese Medicine and Pharmacy, Department of Health. Taipei, Taiwan. China Press, Kuala Lumpur.

[6] Nakaoki T, Morita N (1960). Studies on the medicinal resources XVI Flavonoids of the leaves of Castanea pubinervis Schneid Hydrocotyle wilfordi Maxim Sanguisorba hakusanensis Makino Euptelaea polyandra Sieb et Zucc Carthamus tinctoria L Lactuca repens Maxim Daucus carota L. var. sati. J. Pharm. Soc. Japan.

[7] Shigematsu N, Kouno I, Kawano N (1982). Quercetin 3-O-(6″-caffeoylgalactoside) from Hydrocotyle sibthorpioides. Phytochemistry.

[8] Voigt G, Hiller K, Franke P (1981). On The structure of flavonoids from Hydrocotylevulgaris L. Pharmazie.

[9] Della Greca M, Fiorentino A, Mongoni L, Molinara A, Monaco P, Previtera L (1993). Cytotoxic 9, 10-Dihydrophenanthrenes from Juncus effuses L. Tetrahedron.

[10] Kwon HC, Zee OP, Lee KR (1998). Two New Monogalactosylacylglycerols from Hydrocotyle ramiflora. Planta Med..

[11] Rocha FF, Almeida CS, Santos RT, Santana SA, Costa EA, Paula JR, Vanderlinde FA (2011). Anxiolytic-like and sedative effects of Hydrocotyle umbellata L Araliaceae, extract in mice. Rev. Bras. Farmacogn..

[12] Godfrey RK, Wooten JW (1981). Aquatic and wetland plants of southeastern United State: dicotyledons. University of Georgia Press, Athens, Georgia.

[13] Mohlenbrock RH (2008). Acanthaceae to Myricaceae:Water Willows to Wax Myrtles. 1st ed. SIU Press, Carbondale, Illinois, US.

[14] Reis H, Gomes L, Freitas M, Nogueira JO, Silva E, Maranha O, Carneiro D (1992). Como utilizar plantas medicinais. Sistema Único de Saúde- Ministério da Saúde, Goiânia.

[15] Florentino F, Nascimento MVM, Galdino PM, Brito AF, Rocha FF, Tonussi CR, Lima TCM, Paula JR, Costa EA (2013). Evaluation of analgesic and anti-inflammatory activities of Hydrocotyle umbellata L Araliaceae (acariçoba) in mice. An. Acad. Bras. Cienc..

[16] OECD (2001). OECD Guideline for Testing of Chemicals No 425: Acute Oral Toxicity: Up-and-Down Procedure. Organisation for Economic Cooperation and Development, Paris.

[17] Winter CA, Risley EA, Nuss GW (1962). Carrageenan-induced edema in hind paw of the rat as an assay for anti-inflammatory drugs. Exp. Biol. Med..

[18] Matsumoto K, Obara S, Kuroda Y, Kizu J (2015). Anti-inflammatory effects of linezolid on carrageenan-induced paw edema in rats. J. Infect. Chemother..

[19] Druckerei CH (2002). European Pharmacopœia.

[20] Geissman TA (1962). The Chemistry of Flavonoid Compounds.

[21] Stahl E (1996). Thin Layer Chromatography.

[22] Wagner H, Baldt S, Zagainiski E (1983). Droger analyse.

[23] Mabry JT, Markham KR, Thomas MB (1970). The Systemic Identification of Flavonoids 2nd ed.

[24] Morris CJ (2003). Carrageenan-induced paw edema in the rat and mouse. Methods Mol. Biol..

[25] Shin S, Jeon JH, Park D, Jang JY, Joo SS, Hwang BY, Choe SY, Kim YB (2009). Anti-inflammatory effects of an ethanol extract of Angelica gigas in a Carrageenan-air pouch inflammation model. Exp. Anim..

[26] Srinivasan K, Muruganandan S, Lal J, Chandra S, Tandan S, Prakash VR (2001). Evaluation of anti-inflammatory activity of Pongamia pinnata leaves in rats. J. Ethnopharmacol..

[27] Mahomoodally MF, Gurib-Fakim A, Subratty AH (2005). Antimicrobial activities and phytochemical profiles of endemic medicinal plants of Mauritius. Pharm. Biol..

[28] Visioli F, Poli A, Gall C (2002). Antioxidant and other biological activities of phenols from olives and olive oil. Med. Res. Rev..

[29] Kumar S, Pandey AK (2013). Chemistry and biological activities of flavonoids: an overview. Sci. World. J..

[30] Zhang L, Ravipati AS, Koyyalamudi SR, Jeong SC, Reddy N, Smith PT, Wu MJ (2011). Antioxidant and anti-inflammatory activities of selected medicinal plants containing phenolic and flavonoid compounds. J. Agric. Food Chem..

[31] da Silva Oliveira C, Fonseca Maciel L, Spínola Miranda M, da Silva Bispo E (2011). Phenolic compounds, flavonoids and antioxidant activity in different cocoa samples from organic and conventional cultivation. Br. Food J..

[32] Agrawal PK (1989). Carbon-13 NMR of Flavonoids: Studies in Organic Chemistry Series, no.39.

[33] Park BJ, Matsuta T, Kanazawa T, Park CH, Chang KJ, Onjo M (2012). Phenolic compounds from the leaves of Psidium guajava II Quercetin and its glycosides. Chem. Nat. Compd..

[34] Yan X, Murphy BT, Hammond GB, Vinson JA, Neto CC (2002). Anti-oxidant activities and antitumor screening of extracts from cranberry fruit (Vacciniummacrocarpon). J. Agric. Food Chem..

[35] Farag MA, El-Ahmady SH, Elian FS, Wessjohann LA (2013). Metabolomics driven analysis of Artichoke leaf and its commercial products via UHPLC–q-TOF-MS and chemometrics. Phytochemistry.

[36] Nakatani N, Kayano SI, Kikuzaki H, Sumino K, Katagiri K, Mitani T (2000). Identification, quantitative determination and antioxidative activities of chlorogenic acid isomers in Prune (Prunus domestica L ). J. Agric. Food Chem..

[37] Hyun K, Min BS, Choi JS (2010). Isolation of phenolics, nucleosides, saccharides and an alkaloid from the root of Aralia cordata. Nat. Prod. Sci..

[38] Pauli GF, Kuczkowiak U, Nahrstedt A (1999). Solvent effects in the structure dereplication of caffeoyl quinic acids. Magn. Reson. Chem..

[39] Rogerio AP, Dora CL, Andrade EL, Chaves JS, Silva LF, Lemos-Senna E, Calixto JB (2010). Anti-inflammatory effect of quercetin-loaded microemulsion in the airways allergic inflammatory model in mice. Pharmacol. Res..

[40] Boots AW, Wilms LC, Swennen EL, Kleinjans JC, Bast A, Haenen GR (2008). In- vitro and ex-vivo anti-inflammatory activity of quercetin in healthy volunteers. Nutr..

[41] Comalada M, Camuesco D, Sierra S, Ballester I, Xaus J, Gálvez J, Zarzuelo A (2005). In-vivo quercitrin anti-inflammatory effect involves release of quercetin, which inhibits inflammation through down-regulation of the NF-κB pathway. Eur. J. Immunol..

[42] Guardia T, Rotelli AE, Juarez AO, Pelzer LE (2001). Anti-inflammatory properties of plant flavonoids Effects of rutin, quercetin and hesperidin on adjuvant arthritis in rat. Farmaco.

[43] Hwang SJ, Kim YW, Park Y, Lee HJ, Kim KW (2014). Anti-inflammatory effects of chlorogenic acid in lipopolysaccharide-stimulated RAW 264 7 cells. Inflamm. Res..

[44] Dos Santos MD, Almeida MC, Lopes NP, De Souza GE (2006). Evaluation of the anti-inflammatory, analgesic and antipyretic activities of the natural polyphenol chlorogenic acid. Biol. Pharm. Bull..

